# Hematoma Prediction in Gender-Affirming Mastectomies: A Single-Surgeon Experience with 267 Patients

**DOI:** 10.3390/jcm14134656

**Published:** 2025-07-01

**Authors:** Yoram Wolf, Ron Skorochod

**Affiliations:** 1 Unit of Plastic and Reconstructive Surgery, Hillel Yaffe Medical Center, Hadera 38100, Israel; yoramw@hymc.gov.il; 2 Rappaport Faculty of Medicine, Technion Institute of Technology, Haifa 320002, Israel

**Keywords:** hematoma, gender-affirming surgery, complications

## Abstract

**Background/Objectives:** Gender-affirming mastectomies are a pivotal step in the gender-affirmation process. These procedures represent the concordance between an individual’s appearance, as seen by the environment, and his/her perception of themselves. Hematomas are a growing concern in gender-affirming mastectomies, as they carry the risk for reoperation, increased length of hospital stay, and sub-par aesthetic outcomes. Recognition of factors contributing to the development of hematomas in gender-affirming mastectomies can improve surgical outcomes and patient satisfaction. In this study, we hope to shed light on variables potentially contributing to the development of post-operative hematomas in our experience with 267 gender-affirming mastectomies. **Methods:** Medical records of 267 consecutive gender-affirming mastectomies performed by the senior author were included in this study. Relevant demographic, clinical, and surgical characteristics were collected from patients’ medical files. The patients were stratified based on whether they developed post-operative hematomas. Univariate and multivariate analyses were performed to determine the impact of various factors on the risk of the development of post-operative hematomas. **Results:** The study groups were found to be similar in most baseline demographic and surgical characteristics. Statistically significant differences were seen regarding mean BMI, use of combined TRT and estrogen blockers, surgical technique, previous reduction mammaplasty, and intra-operative tissue resection weight (*p*-value = 0.007, 0.03, <0.001, 0.02, <0.001). Multivariate logistic regression was performed to predict post-operative hematomas. The covariates in question were statistically significant variables that differed between the groups. Previous reduction mammaplasty was found to be a statistically significant independent predictor of post-operative hematomas, with an OR of 41.55 (95% CI 4.2–408.3), and the “free NAC” surgical technique was found to decrease the incidence of post-operative hematomas, with an OR of 0.015 (95% CI 0.003–0.064). **Conclusions:** A history of reduction mammaplasty is a substantial risk factor for the development of post-operative hematomas in gender-affirming mastectomies. Of the various surgical techniques, the use of the “free NAC” technique can, to some degree, reduce the risk of hematoma development.

## 1. Introduction

Gender-affirming mastectomy, or chest masculinization surgery, is a surgical procedure that is conducted with the purpose of aligning an individual’s physical appearance with their gender identity [1,2]. During the procedure, resection of all or part of the breast tissue is performed to create a flat and masculine chest contour [3]. The procedure often represents a fundamental aspect of gender-affirming care for gender diverse individuals and is typically regarded as a crucial and pivotal step in the affirmation process [4,5]. The result is not merely a physical transformation but is also accompanied by a notable enhancement in psychological well-being and quality of life [6].

The main indication for gender-affirming mastectomy is a recorded chest dysphoria, as defined by the DSM. Chest dysphoria refers to distress resulting from a discrepancy between an individual’s gender identity and the physical appearance of their chest. It is often recommended that surgical measures follow medical and psychological interventions to create a holistic approach that best serves the individual’s needs and desires [7,8,9]. In some countries, surgical intervention must follow prolonged therapy with hormone replacement to ensure patients’ desire and gender concordance.

Over the past years, a notable increase has been observed in the demand for gender-affirming procedures. The trend may be, in part, attributed to improved social awareness, greater accessibility to gender-affirming care, and a growing body of evidence supporting the effectiveness and safety of these interventions [10,11]. With more institutions adopting standardized protocols and multidisciplinary teams, outcomes have become increasingly predictable, and patient satisfaction remains high.

Despite the apparent psychosocial benefits of gender-affirming mastectomies, as with all surgical procedures, they carry a risk for potential complications. While most patients report favorable outcomes, some may experience complications, such as delayed wound healing, infection, seroma, hematoma, and even skin and nipple areola complex (NAC) necrosis. Since the procedure is considered an elective and cosmetic surgical procedure, it is crucial to establish its safety and define potential risk factors for complications.

Hematomas are amongst the most common complications in all surgical procedures. They may result in subsequent impaired wound healing, extended hospital stays, and the need for revision surgeries [12,13]. The gender-diverse population that opts for chest masculinization surgery is a potential risk group because of the use of hormonal replacement and suppression therapy [14].

In this retrospective cohort study, we examined the medical records of all patients who underwent chest masculinization surgeries by the senior author. Our goal was to determine potential risk factors for the development of hematomas in the post-operative period and quantify their impact. We believe that our large-scale experience in the procedure can shed light on this crucial topic.

## 2. Materials and Methods

### 2.1. Ethical Considerations

This study was conducted after obtaining the appropriate approval from the local institution’s Institutional Review Board (IRB). The researchers adhered meticulously to the approved research protocol. In instances of ethical queries, the IRB was directly contacted for guidance. All data were handled in compliance with institutional and international data protection and privacy regulations, including anonymization of identifiable patient information. Given the retrospective design, individual patient consent was waived by the IRB.

### 2.2. Data Collection

All patients who underwent gender-affirming mastectomies by the senior author (Y.W.) during the study period were eligible for participation. The study period spanned from 2003 to 2023, allowing for a comprehensive review of consecutive cases. Patients were excluded in instances of incomplete medical records or non-adherence to a minimal follow-up period, which was defined as at least 30 days from the date of surgery. Following a thorough review of electronic medical records and operative reports, relevant demographic, clinical, and surgical characteristics were extracted using a standardized data abstraction form.

Demographic characteristics included patients’ age, body mass index (BMI), and presence of comorbidities, such as hypertension, diabetes mellitus, hypothyroidism, asthma, psychiatric disorders, and smoking status. Clinical and surgical variables included current hormonal therapy (e.g., testosterone), surgical technique, operative time, intra-operative estimated blood loss, resection weight, liposuction volume, drain use, and surgery-related complications. Complications were categorized as either minor (managed conservatively; Clavien–Dindo grades I–II) or major (requiring surgical intervention under local or general anesthesia; Clavien–Dindo grade III). Wound-specific issues, such as hematoma, seroma, dehiscence, infection, and nipple–areola complex (NAC) necrosis, were also specifically recorded.

### 2.3. Statistical Analysis

Statistical analysis was performed using statistical software (SPSS Version 29.0, IBM Corp., Chicago, IL, USA). Continuous variables were expressed as the mean alongside the corresponding standard deviation (SD), and categorical variables were expressed as frequency and percentage of the entire cohort. Categorical variables were compared using the chi-square test or Fisher’s exact test, as appropriate, and continuous variables were analyzed using Student’s *t*-test (for normally distributed data) or the Mann–Whitney U test (for non-normally distributed data).

To determine associations between patient or surgical characteristics and the primary outcome (post-operative hematoma), univariate analysis was first conducted. Variables that achieved a *p*-value < 0.05 in the univariate analysis were subsequently entered into a multivariate binary logistic regression model, with hematoma formation as the dependent variable. Adjusted odds ratios (ORs) and 95% confidence intervals (CIs) were calculated to assess the strength and significance of associations.

### 2.4. Surgical Techniques

This trial describes our experience with four main surgical approaches: the peri-areolar approach, omega-shaped resection (nipple–areola complex on scar), spindle-shaped mastectomy with inferior NAC flap, and spindle-shaped mastectomy with a free NAC graft.

The peri-areolar approach involves cutting around and reducing the areola, followed by the creation of a dermal flap to support the nipple–areola complex. The breast tissue is dissected through this incision, and redundant skin is tightened by concentric suturing.

The omega-shaped resection extends the incision medially and laterally into an omega (Ω) shape. This allows for a broader resection of skin and glandular tissue. Closure results in a horizontal scar at the level of the nipple, facilitating improved contouring in patients with moderate skin excess.

The spindle-shaped mastectomy with an inferior NAC flap preserves a 2 mm thick inferiorly based skin flap on which the NAC is based. A round opening is made in the superior skin flap at the ideal nipple position, and the NAC is inset through this window. The spindle-shaped excision is then sutured, resulting in a horizontal scar.

In the free NAC technique, the entire NAC is removed as a full-thickness graft following a complete spindle mastectomy. After de-epithelialization of the graft site, the NAC is repositioned and secured as a free skin graft. This method is often preferred in patients with significant breast size or ptosis.

## 3. Results

A total of 267 patients met the inclusion criteria and were included in the final analysis. The overall incidence of post-operative hematomas in this cohort was 12% (n = 32). Patients were stratified into two groups based on the occurrence of hematomas. Comparative analysis of baseline demographic and surgical characteristics between patients who developed hematomas and those who did not revealed that the groups were generally similar across most parameters.

However, statistically significant differences were identified in several variables. The patients who developed hematomas had a significantly lower mean body mass index (BMI) compared to those who did not (*p* = 0.007). The use of combined testosterone replacement therapy (TRT) and estrogen blockade was more prevalent among hematoma patients (*p* = 0.03). Differences were also observed in surgical technique, with the “free NAC” approach being significantly less common among the patients who developed hematomas (*p* < 0.001). Additionally, the patients with a history of prior reduction mammaplasty were more likely to develop hematomas (*p* = 0.02), and the mean intra-operative tissue resection weight was significantly higher in the hematoma group (*p* < 0.001) (Table 1).

To identify independent predictors of hematoma formation, a multivariate logistic regression analysis was performed. The model included all variables that were found to be statistically significant in the univariate comparisons. A history of previous reduction mammaplasty emerged as a strong independent risk factor, with an odds ratio (OR) of 41.55 (95% CI, 4.2–408.3; *p* < 0.001). In contrast, the use of the “free NAC” surgical technique was associated with a significantly lower risk of hematoma, with an OR of 0.015 (95% CI, 0.003–0.064; *p* < 0.001). Full multivariate regression results are presented in (Table 2).

## 4. Discussion

Hematomas represent one of the most prevalent complications associated with gender-affirming mastectomies, consistently representing the primary indication for reoperation [15,16]. A substantial body of literature has sought to identify definitive factors associated with the formation of hematomas in the post-operative period following gender-affirming mastectomies, with contradicting results [17,18,19,20,21,22]. Bekisz et al. [23] performed a systematic literature review to identify variables supported by the literature as being associated with hematoma in gender-affirming mastectomies. The authors concluded that there is sufficient evidence to support a positive association between smoking and hematoma formation. Conversely, free nipple grafting proved to decrease the incidence of hematoma formation. On the contrary, obesity, prior breast reduction, weight of the mastectomy flap, testosterone replacement therapy, and systemic tranexamic were not found to impact hematoma incidence.

The impact of free nipple grafting on the rate of adverse events and revision surgeries has been a source of debate. While some research advocates its protective effect, others highlight the potential danger of the technique. Wilson et al. [24] performed a meta-analysis focusing on the outcomes of various techniques for chest masculinization. They reported that in their analysis, free nipple grafting resulted in significantly fewer revision surgeries and second surgeries as compared to other techniques. Opposite results were reported by Cuccolo et al. [25], who conducted a retrospective cohort study of nearly 600 patients from the ACS-NSQIP. They found that the rate of reoperation for hematoma was almost double in the group of free nipple grafting, with no difference in the number of overall complications between the groups.

In our study, we postulated that a lower BMI, the surgical technique, previous reduction mammaplasty, the combination of TRT and estrogen blockers, and the weight of intra-operative tissue resection may be potential contributors to hematoma development. After adjusting for potential confounding variables, previous reduction mammaplasty emerged as an independent significant risk factor for hematoma development, with the risk increasing by a factor of 40. Furthermore, the utilization of the “free NAC” technique was identified as an independent factor that reduced the risk of hematoma by 85%.

Salim et al. [26] presented their findings from a case series of five patients who underwent gender-affirming mastectomies following prior breast reduction. In their experience, the average blood loss was estimated to be 42 mL, and no adverse events were recorded.

In their practice, Cyril et al. employed a staged mastectomy approach for oncological indications, with patients undergoing breast reduction surgery prior to performing a nipple-sparing mastectomy. The objective was to guarantee adequate vascularization of the NAC and thereby reduce the risk of ischemia. In their experience, prior reduction was found to have no impact on the risk of hematoma development while ensuring optimal aesthetic outcomes and NAC vascularization.

Nevertheless, to date, no large-scale cohort studies have been published in the literature regarding the impact of previous reduction mammaplasty on the surgical outcomes of gender-affirming mastectomies specifically.

In our study, this factor was identified as a critical risk factor; therefore, further research should be conducted to explore this association.

It is worthwhile to mention the lack of statistical impact of hormonal therapy on hematoma formation, as per the multivariate regression model. Despite the notable differences in prevalence observed in the univariate analysis, the results did not reach significance in the multivariate model. Although the explanation may indeed stem from a lack of significant impact when other confounders were analyzed in the model, it could also be the result of our systemic institutional practice of hormonal therapy cessation 14 days prior to surgery, thus decreasing the bioavailability of hormones and potentially minimizing their impact on surgical outcomes.

While our study offers valuable insights from a large cohort and a single surgeon’s experience, it is not without limitations.

It should be noted that this is a retrospective cohort study, which precludes the possibility of differentiating between causality and association. Consequently, further prospective studies are required to validate the conclusions of our data analysis in terms of clinical prediction abilities.

Furthermore, the conclusions drawn in this study are based on a relatively small sample size of hematomas that occurred in this cohort. Although it provided a valuable foundation for initial conclusions and hypotheses, larger-scale studies are necessary to establish the association with greater certainty.

In this context, it is especially crucial to acknowledge the potential overfitting of the multivariate regression model that stems from the low prevalence of hematomas in our cohort. This potential concern, alongside the instability of outcome prediction, is highlighted by the extremely wide confidence interval.

Finally, although the single-surgeon experience eliminates many of the potential confounding factors related to the provider, it is reasonable to anticipate a learning curve over time. It is plausible that the earlier cases reflect a relative lack of experience compared to the more recent cases. Upon analysis of the yearly incidence of hematomas in the cohort, we found slight variations that did not reach statistical significance, most probably due to the overall low number of hematomas observed in the cohort.

In conclusion, the results of our study demonstrate that previous reduction mammaplasty is an independent significant risk factor for hematomas in gender-affirming mastectomy. Conversely, the utilization of the “free NAC” technique has the potential to mitigate the risk and is an independent risk factor.

Additional large-scale research is essential to validate the nature of this association and to explore other potential risk factors. It is our hope that this research will prove useful in the future development of risk stratification formulas that consider the effect of individual variables on the clinical course.

## Figures and Tables

**Table 1:** Baseline characteristics of the study cohort, grouped by the development or absence of post-operative hematomas.

Variable	No Post-Operative Hematoma (n = 235)	Post-Operative Hematoma (n = 32)	*p*-Value
Age, mean ± STD	21.1 ± 6.5	22.6 ± 8.2	0.24
BMI, mean ± STD	25.9 ± 5.5	22.4 ± 4.7	0.007
Testosterone Therapy (TRT)	104	14	0.97
Estrogen Blockers	6	1	0.85
TRT + Estrogen Blockers	37	10	0.03
Hemoglobin (g/dL)	13.9 ± 1.5	13.9 ± 1.3	0.97
Platelets	275.3 ± 60.4	265.1 ± 62.7	0.39
Surgery			<0.001
- Peri-areolar.	7	26	
- NAC on scar.	5	0	
- NAC flap.	53	0	
- Free NAC.	170	6	
Hypertension	1	0	0.71
Diabetes Mellitus	3	0	0.52
Previous Reduction Mammaplasty	2	2	0.02
Resection Weight, mean ± STD	519.1 ± 342.8	228.3 ± 166.4	<0.001
Liposuction Volume, mean ± STD	222.7 ± 307.6	140.6 ± 248.5	0.31

**Table 2:** Results of a multivariate logistic model aimed at the prediction of post-operative hematoma, accounting for statistically significant variables.

Variable	Correlation Coefficient	Standard Error	Odds Ratio	95% Confidence Interval	*p*-Value
Resection Weight	−0.003	0.002	0.997	0.994–1.001	0.145
Previous Reduction Mammaplasty	3.73	1.17	41.55	4.23–408.34	0.001
TRT + Estrogen Blockers	−0.47	0.74	0.63	0.15–2.64	0.52
Surgical Method					
- Peri-areolar.					
- NAC on scar.	−22.34	17,681.2			0.999
- NAC flap.	−22.26	5445.05			0.997
- Free NAC.	−4.23	0.759	0.015	0.003–0.064	<0.001

## Data Availability

The original contributions presented in this study are included in this article. Further inquiries can be directed to the corresponding author.
